# Alanyl-tRNA Synthetase Quality Control Prevents Global Dysregulation of the Escherichia coli Proteome

**DOI:** 10.1128/mBio.02921-19

**Published:** 2019-12-17

**Authors:** Paul Kelly, Nicholas Backes, Kyle Mohler, Christopher Buser, Arundhati Kavoor, Jesse Rinehart, Gregory Phillips, Michael Ibba

**Affiliations:** a The Ohio State University Molecular, Cellular and Developmental Biology Program, The Ohio State University, Columbus, Ohio, USA; b Department of Veterinary Microbiology & Interdepartmental Microbiology Graduate Program, Iowa State University, Ames, Iowa, USA; c Department of Cellular & Molecular Physiology, Yale School of Medicine, New Haven, Connecticut, USA; d Systems Biology Institute, Yale University, New Haven, Connecticut, USA; e Oak Crest Institute of Science, Monrovia, California, USA; f Center for RNA Biology, The Ohio State University, Columbus, Ohio, USA; g Department of Microbiology, The Ohio State University, Columbus, Ohio, USA; Vanderbilt University

**Keywords:** aminoacyl-tRNA, errors, protein synthesis, quality control, translation, tRNA

## Abstract

Mechanisms have evolved to prevent errors in replication, transcription, and translation of genetic material, with translational errors occurring most frequently. Errors in protein synthesis can occur at two steps, during tRNA aminoacylation and ribosome decoding. Recent advances in protein mass spectrometry have indicated that previous reports of translational errors have potentially underestimated the frequency of these events, but also that the majority of translational errors occur during ribosomal decoding, suggesting that aminoacylation errors are evolutionarily less tolerated. Despite that interpretation, there is evidence that some aminoacylation errors may be regulated, and thus provide a benefit to the cell, while others are clearly detrimental. Here, we show that while it has been suggested that regulated Thr-to-Ser substitutions may be beneficial, there is a threshold beyond which these errors are detrimental. In contrast, we show that errors mediated by alanyl-tRNA synthetase (AlaRS) are not well tolerated and induce a global stress response that leads to gross perturbation of the Escherichia coli proteome, with potentially catastrophic effects on fitness and viability. Tolerance for Ala mistranslation appears to be much lower than with other translational errors, consistent with previous reports of multiple proofreading mechanisms targeting mischarged tRNA^Ala^. These results demonstrate the essential role of aminoacyl-tRNA proofreading in optimizing cellular fitness and suggest that any potentially beneficial effects of mistranslation may be confined to specific amino acid substitutions.

## Abstract (precis)

Errors in protein synthesis have historically been assumed to be detrimental to the cell. While there are many reports that translational errors are consequential, there is a growing body of evidence that some mistranslation events may be tolerated or even beneficial. Using two models of mistranslation, we compare the direct phenotypic effects of these events in Escherichia coli. This work provides insight into the threshold for tolerance of specific mistranslation events that were previously predicted to be broadly neutral to proteome integrity. Furthermore, these data reveal the effects of mistranslation beyond the general unfolded stress response, leading to global translational reprogramming.

## INTRODUCTION

Throughout all domains of life, mechanisms have evolved to minimize errors in protein synthesis. Translational fidelity is maintained at two distinct steps of protein synthesis, surveillance of accurate aminoacyl-tRNA (aa-tRNA) pairing and cognate A site tRNA recognition during ribosome decoding (1). While it was previously shown that translational errors occur more frequently than do errors in replication and transcription, the extent of these errors had not been well defined. Through advances in proteome-wide mass spectrometry, the prevalence of certain translational errors has been extensively characterized (2). Observations from these efforts suggest that errors in ribosomal decoding by near-cognate anticodons are far more frequent than are errors likely caused by misaminoacylated tRNAs. This result suggests that errors in decoding are evolutionarily better accommodated than are aminoacyl-tRNA errors which can occur at higher frequencies if unchecked by proofreading.

Accurate aa-tRNA pairing is maintained by aminoacyl-tRNA synthetases (aaRSs). These enzymes are responsible for pairing free amino acids in the cell and ligating them onto their cognate tRNAs (3). aaRSs perform their function in two distinct steps. First, free amino acid are activated in an ATP-dependent manner, forming an aminoacyl adenylate. Upon amino acid activation, the amino acid is transferred onto its cognate tRNA, which can then be released to be used in translation. The Escherichia coli genome contains 20 aaRS genes, one for each of the proteinogenic amino acids. As a result of the shared chemicophysical properties of many amino acids, half of the aaRS enzymes can potentially misactivate numerous noncognate amino acids (reviewed in reference 4). To prevent erroneous translation, aaRSs have evolved proofreading mechanisms to prevent misactivated amino acids from being transferred onto tRNAs and subsequently released to the translation machinery for protein synthesis. aaRS-catalyzed proofreading mechanisms (commonly referred to as “editing”) can occur immediately following amino acid activation in which the aminoacyl adenylate will be hydrolyzed, releasing the amino acid back into the pool of free metabolites. For example, IleRS utilizes pretransfer proofreading to prevent Val-AMP from being transferred onto tRNA^Ile^ (5). Alternatively, some aaRS genes encode a second, distinct catalytic active site to monitor aminoacyl moieties following the transfer onto the 3′ end of the tRNA. The aforementioned mechanism of posttransfer proofreading is widespread and has been well characterized for several aaRSs to discriminate noncognate amino acids, including Tyr-tRNA^Phe^ (6), Nva-tRNA^Ile/Leu^ (7, 8), Ser-tRNA^Thr^ (9), and Ser-tRNA^Ala^ (10, 11). In addition to proofreading activities by the aaRS, several free-standing enzymes are genomically encoded which have activity on misaminoacylated tRNA species following release by the aaRS. Some of the more widely characterized *trans*-editing factors are members of the INS-like family of enzymes that share similar proofreading active-site architecture to the prolyl-tRNA synthetase enzymes (12). Additionally, free-standing alanyl-tRNA synthetase (AlaRS) proofreading domains are present among the AlaXP family of enzymes. AlaXP is an evolutionarily conserved factor which contributes to the accuracy of tRNA^Ala^ aminoacylation. Interestingly, E. coli is an outlier among most organisms in that it does not encode an AlaXP homolog (13). The absence of this factor makes E. coli a strong model for studying AlaRS mistranslation, as there is not a redundant mechanism to correct Ser-tRNA^Ala^ product formation. Recently, a novel *trans*-editing factor, ANKRD16, was identified in vertebrates which binds to Ser-AMP in complex with AlaRS, preventing the transfer onto tRNA^Ala^ (14). Finally, the d-tyrosyl deacylase (DTD) whose function was originally characterized to prevent d-amino acid aminoacylation was found to have proofreading activity against Gly-tRNA^Ala^ (15). Taken together, the redundancy in proofreading factors, specifically those whose activity is to prevent erroneous tRNA^Ala^ aminoacylation, highlights the potential cost of alanine mistranslation events.

In addition to the biochemical identification and characterization of redundant tRNA^Ala^ proofreading factors, the physiological cost of AlaRS errors has been described in higher eukaryotic model organisms. A mutant AlaRS allele (*sti*) in mice was shown to lead to neurodegeneration (16) and cardioproteinopathy (17) due to the accumulation of misfolded proteins. Interestingly, *in vitro* characterization of the mutant AlaRS protein showed only partial loss of proofreading activity compared to the wild-type enzyme, suggesting that low-frequency AlaRS errors are costly to the mammalian proteome. Furthermore, recapitulation of the *sti* allele into the mitochondrial AlaRS led to embryonic lethality (18), suggesting that the mitochondrial proteome is even more intolerant to AlaRS errors.

Despite the importance for AlaRS proofreading and the presumed negative impact on proteome homeostasis of Ala mistranslation events, evidence for beneficial mistranslation has also recently been observed. During oxidative stress, a critical cysteine in the E. coli threonyl-tRNA synthetase (ThrRS) proofreading site becomes oxidized, leading to an overall decrease in ThrRS fidelity (19). Additionally, oxidative stress causes elevated mismethionlyation on noncognate tRNAs in both bacteria and eukaryotes, which serves as a protective mechanism against reactive oxygen species (20, 21). In addition to cysteine oxidation, it was recently identified during a screen for aaRS acetylation that ThrRS can be posttranslationally acetylated at K169, leading to a decrease in ThrRS accuracy (22). Taken together, it appears that during protein synthesis, specific translational errors may be regulated and provide some benefit for the cell under certain environmental conditions. While recent advances in proteome mass spectrometry have led to greater quantification of mistranslational errors, the physiological consequences of these errors have not been extensively explored.

Here, we report the global consequences of translational errors in E. coli on cellular physiology and fitness. Despite recent evidence that elevated ThrRS-mediated mistranslation errors occur during both oxidative stress and a regulated protein acetylation event, we show that high levels of serine misincorporation at threonine codons are detrimental to E. coli. Furthermore, we show that growth defects caused by AlaRS-mediated errors are not only due to the accumulation of mistranslated proteins but rather to a gross perturbation to proteome homeostasis.

## RESULTS

### High levels of serine miscoding are toxic to E. coli.

**FIG 1:**
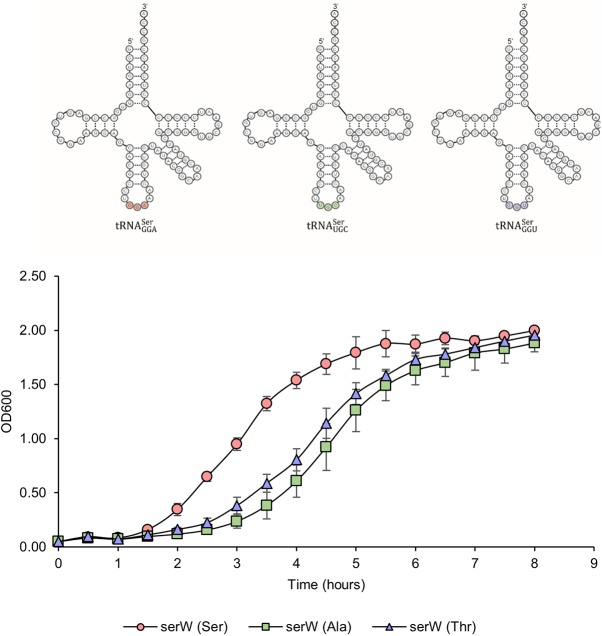
Serine miscoding is toxic in E. coli. Chimeric tRNA^Ser^ variants were generated to decode at either the Ser (anticodon, GGA), Ala (UGC), or Thr (GGU) codon (top). Mutant tRNAs were expressed in wild-type E. coli, and growth was monitored (bottom). Both tRNA mutants caused growth defects in MG1655. All growth experiments were performed in triplicate, and error bars indicate the standard deviation of the replicates.

Previous independent reports studying the effects of translation errors suggest that uncoded Thr-to-Ser substitutions are better tolerated by the cell than are Ala-to-Ser substitutions (10, 19). It can be speculated that the cause for this difference is the shared terminal hydroxyl group among threonine and serine functional groups leading to a more conservative substitution. Given that ThrRS-mediated mistranslation may be upregulated during cellular stresses such as elevated reactive oxygen species, we wanted to determine the tolerance for serine mistranslation in E. coli. To study the substitution-specific effects of mistranslation, wild-type tRNA^Ser^ or tRNA^Ser^ mutants which decode at either alanine or threonine codons were expressed in wild-type E. coli. Plasmid constructs expressing tRNA^Ser^ variants were generated by cloning the entire *serW* transcription unit which carries one of the five tRNA^Ser^ genes (Ser5) (23) into a low-copy-number plasmid under the control of the native *serW* promoter and terminator, allowing for reliable tRNA processing. Based on previous reports, *serW* abundance (24) and aminoacylation levels (25) are similar to those of other tRNA^Ser^ isoacceptors, providing a good model for studying global serine mistranslation events. This approach has previously been used to show that Ser-to-Ala mistranslation led to elevated tumorigenesis in mice (26). When miscoding tRNA^Ser^ mutants were expressed in E. coli, they led to a similar decrease in growth rate compared to exogenously expressed wild-type tRNA^Ser^ (Fig. 1). The results from this experiment indicate that elevated levels of serine miscoding, regardless of predicted translational error, will lead to an overall growth defect compared to wild-type E. coli when grown in rich medium.

### AlaRS proofreading is required for optimal growth in E. coli.

To compare the cost of Ala-to-Ser versus Thr-to-Ser mistranslation events in E. coli, previously characterized mutations in both the AlaRS and ThrRS proofreading domains were made in isogenic MG1655 genetic backgrounds. Architecturally, these enzymes are predicted to share similar proofreading domains (10), both of which have critical cysteine residues in the active site that coordinate the 3′ end of the tRNA for noncognate hydrolysis (9, 11). *In vitro* experimentation has shown that mutations of these cysteine residues to alanine will partially eliminate the proofreading activity of these enzymes (9, 10). Through the use of a mass spectrometry-based reporter, it was shown that upon mutation of C182 in ThrRS, there was an increase in Thr-to-Ser substitutions, but these mistranslation events had no effect on cell viability (27). In comparison, using a temperature-sensitive *alaS* allele and AlaRS variants expressed on low-copy-number plasmids, it was shown that when C666 (homologous to C182 in ThrRS) was mutated to alanine, E. coli was sensitized to noncognate serine stress (10). Because these independent efforts utilized different genetic approaches and growth conditions, we sought to generate isogeneic E. coli aaRS mutants to directly compare the phenotypic cost of low-level serine mistranslation at both alanine and threonine codons. *In vitro* kinetic analyses of the corresponding proofreading-defective ThrRS and AlaRS variants showed that despite serine acting as a noncognate substrate for amino acid activation, serine is a poor substrate for activation compared to Thr and Ala, respectively. This would suggest that any phenotypes associated with these variants would be representative models for studying the effects of low-frequency translation errors (19, 28).

**FIG 2:**
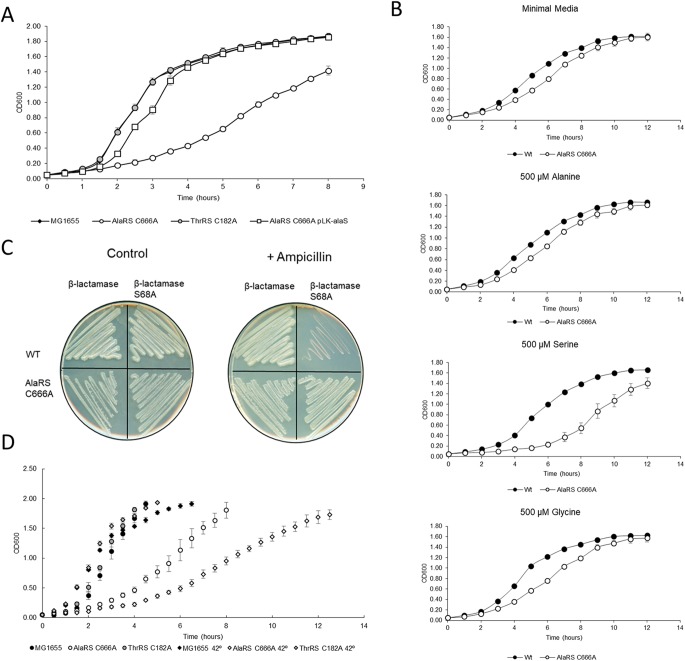
AlaRS fidelity is required for optimal growth in E. coli. (A) AaRS mutants and the AlaRS C666A complement strain (pLK-*alaS*) were grown in LB, and their growth was monitored. In rich medium, AlaRS fidelity is required for optimal growth. (B) AlaRS C666A was grown in M9 minimal medium supplemented with exogenous amino acids to determine the toxicity of noncognate stress. (C) Serine mistranslation was observed in the AlaRS C666A mutant using a β-lactamase S68A mistranslation reporter. (D) The severity of the AlaRS C666A growth defect was enhanced when grown at 42°C. All growth experiments were performed in triplicate, and error bars indicate the standard deviation of the replicates. WT, wild type.

In rich medium, the AlaRS C666A variant had a marked decrease in growth compared to wild-type E. coli (Fig. 2A). This result was unexpected, as this strain is neither starved for cognate alanine nor supplemented with excess serine. As serine is a poor substrate for AlaRS activation, we would predict a highly accurate Ala-tRNA^Ala^ pool in the AlaRS C666A strain, suggesting that very low levels of serine misincorporation at alanine codons are detrimental to E. coli. The observed cellular growth defect could be restored by complementing the wild-type *alaS* allele on a low-copy-number plasmid (Fig. 2A). As expected, the ThrRS C182A variant had no change in growth compared to wild-type MG1655 E. coli.

Growth analyses were repeated in minimal medium to determine which noncognate stress is responsible for this defect. As it was previously shown that one of the roles of E. coli DTD is to prevent Gly-tRNA^Ala^ accumulation in the cell (15), glycine supplementation was included in our analyses. While exogenous glycine caused a subtle perturbation to growth, only serine supplementation led to a large growth defect (Fig. 2B). These results suggest that rich medium contains sufficient serine to promote mistranslation at a level resulting in a significant growth defect.

Having confirmed that in the absence of AlaRS editing, serine stress impairs cellular growth, it was important to determine if the serine-specific growth defect correlated with serine mistranslation in the proteome. A similar mass spectrometry-based reporter used to show serine mistranslation in the ThrRS C182A strain (27) was used in the AlaRS mutant. Unfortunately, overexpression of the reporter caused severe cellular growth impairment (data not shown) and was therefore not suitable for *in vivo* analyses. To monitor possible Ala-to-Ser mistranslation *in vivo*, a β-lactamase-based reporter was repurposed from previous work studying Thr-to-Ser mistranslation (29). β-Lactamase is an enzyme responsible for cleaving β-lactam rings, a common class of antibiotic drugs. Within β-lactamase is an essential serine residue that, when mutated, leaves the enzyme inactive and cells unable to grow in the presence of β-lactams (e.g., ampicillin) (30). This essential serine codon was mutated to encode alanine; thus, cells should only be able to grow in the presence of β-lactams if Ala-to-Ser mistranslation occurred at this position. The AlaRS C666A variant, but not the wild type, was able to grow on ampicillin while expressing the β-lactamase S68A variant (Fig. 2C). This result provided direct evidence of AlaRS-mediated serine mistranslation in E. coli. These experiments also strongly suggest that in the absence of AlaRS proofreading, E. coli is sensitive to serine stress, likely due to elevated mistranslation levels.

As the role of heat shock response proteins was expected to be influential in the maintenance of optimal E. coli growth when translational fidelity was perturbed (31), the wild type and ThrRS C182A and AlaRS C666A variants were grown in rich medium at 42°C, and growth was monitored over time. While there was no difference in the ThrRS C182A strain compared to the wild type, the AlaRS C666A variant was further sensitized and impaired in growth at the elevated temperature (Fig. 2D). This observation suggested that the burden caused by mistranslation was likely leading to a global defect preventing the cells from mounting an adequate response to heat stress.

### AlaRS fidelity is required for maintaining proteome homeostasis.

**FIG 3:**
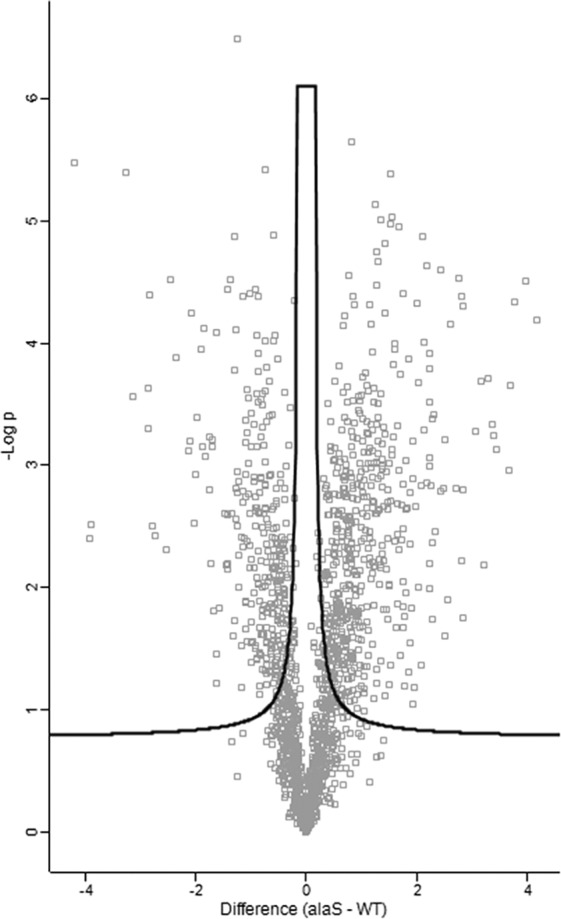
AlaRS-mediated mistranslation disrupts proteome homeostasis. Total proteome analysis was performed on wild-type and AlaRS C666A E. coli. Depicted is a volcano plot of the 833 significantly enriched or underrepresented proteins when AlaRS fidelity is impaired.

Having observed that AlaRS fidelity was required for optimal growth in rich medium and that heat stress further necessitated the requirement for AlaRS aminoacylation accuracy, total proteome analysis was performed to determine which proteins were enriched or underrepresented in the absence of AlaRS proofreading. This analysis gave insight into the array of stress responses that can be influenced by protein mistranslation. In total, 833 proteins were significantly different in the AlaRS C666A variant compared to the wild-type control, with 502 being enriched and 331 being underrepresented (Fig. 3; see also Data Set S1 in the supplemental material). KEGG pathway analysis of the total proteome data set highlights the diverse determinants of cellular homeostasis that translational fidelity can impact. Notably, many of the most enriched pathways are those which are involved in metabolism (Table 1).

**TABLE 1:** KEGG pathway analysis of proteome changes

Description	Observed gene count	Background gene count	False-discovery rate
Enriched pathways			
Metabolic pathways	179	708	1E−18
Biosynthesis of secondary metabolites	107	301	1E−18
Biosynthesis of antibiotics	82	209	4E−16
Biosynthesis of amino acids	60	116	7E−16
Microbial metabolism in diverse environments	75	278	4E−08
Carbon metabolism	40	108	3E−07
Phenylalanine, tyrosine, and tryptophan biosynthesis	13	21	4E−04
Glycine, serine, and threonine metabolism	17	37	4E−04
2-Oxocarboxylic acid metabolism	14	26	4E−04
Cysteine and methionine metabolism	15	32	7E−04
Citrate cycle (TCA cycle)^*a*^	13	27	2E−03
Glutathione metabolism	11	20	2E−03
Histidine metabolism	7	8	4E−03
Bacterial chemotaxis	10	20	6E−03
Oxidative phosphorylation	15	43	6E−03
Methane metabolism	10	26	2E−02
Glyoxylate and dicarboxylate metabolism	13	41	2E−02
Pyruvate metabolism	15	52	2E−02
Vitamin B_6_ metabolism	6	10	2E−02
Sulfur metabolism	11	32	2E−02
Novobiocin biosynthesis	4	4	3E−02
Monobactam biosynthesis	5	8	4E−02
Valine, leucine, and isoleucine biosynthesis	7	16	4E−02
Lysine biosynthesis	6	13	5E−02
Depleted pathways			
Metabolic pathways	103	708	1E−07
Flagellar assembly	17	37	6E−06
Aminoacyl-tRNA biosynthesis	12	25	2E−04
Amino sugar and nucleotide sugar metabolism	15	46	5E−04
Biosynthesis of antibiotics	33	209	6E−03
Glycolysis/gluconeogenesis	12	43	8E−03
Pyruvate metabolism	13	52	9E−03
Fatty acid biosynthesis	6	13	2E−02
Pyrimidine metabolism	14	66	2E−02
Carbon metabolism	19	108	2E−02
Fatty acid metabolism	7	21	3E−02
Oxidative phosphorylation	10	43	3E−02
Purine metabolism	16	91	3E−02
Glycine, serine, and threonine metabolism	9	37	4E−02
Lipopolysaccharide biosynthesis	8	31	4E−02
Alanine, aspartate, and glutamate metabolism	8	32	4E−02
Ribosome	11	56	5E−02

a TCA, tricarboxylic acid.

10.1128/mBio.02921-19.3 DATA SET S1 Total proteome analysis. Changes in protein abundance were monitored in the absence of AlaRS fidelity. Significantly enriched or underrepresented proteins were noted. Download Data Set S1, XLSX file, 0.3 MB. Copyright © 2019 Kelly et al. 2019 Kelly et al. This content is distributed under the terms of the Creative Commons Attribution 4.0 International license.

### Mistranslation disrupts regulation of the translational machinery.

**FIG 4:**
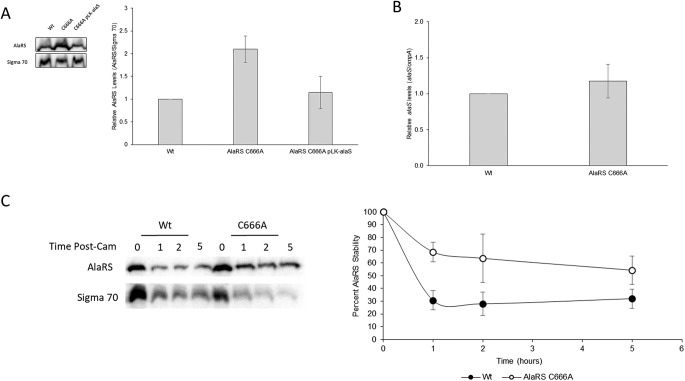
AlaRS fidelity regulates AlaRS protein levels. (A) Representative images (left) and quantification (right) of steady-state AlaRS protein levels in wild-type, AlaRS C666A, and AlaRS C666A-complemented strains. In the absence of AlaRS fidelity, AlaRS protein levels are elevated. (B) qRT-PCR analysis of *alaS* indicates that transcript levels are unaffected in the AlaRS C666A mutant. (C) Representative image (left) and quantification (right) of native AlaRS decay upon treatment of a translation inhibitor. AlaRS protein levels are stabilized when AlaRS fidelity is perturbed. All experiments were performed in triplicate, and error bars indicate the standard deviation of the replicates.

From the KEGG pathway analysis, it was noted that aminoacyl-tRNA biogenesis proteins were underrepresented in the AlaRS C666A proteome. Upon further investigation, it was clear that many aaRS and tRNA modification proteins were depleted in the strain. However, it was particularly interesting to note that AlaRS was ∼2.3× enriched in the AlaRS C666A strain (Data Set S1). To validate this result, steady-state immunoblot analysis was performed to measure AlaRS protein levels in these strains. Recapitulating the proteomic data set, AlaRS protein levels were ∼2× higher in the AlaRS C666A background than in the wild-type strain (Fig. 4A). Interestingly, by complementing the AlaRS C666A strain with a plasmid expressing the wild-type AlaRS gene, the total steady-state AlaRS levels were reduced to a level more similar to the wild-type strain.

AlaRS is known to autoregulate *alaS* transcription through alanine-dependent binding upstream of the *alaS* transcription start site (32). In the presence of high intracellular alanine, AlaRS will repress active *alaS* transcription, presumably leading to a decrease in AlaRS protein levels. As many changes to metabolism were observed, it was of interest to know if perturbation to amino acid biosynthesis was responsible for the increased AlaRS protein levels. To determine if the increase in AlaRS protein levels was due to transcriptional changes to *alaS* expression, quantitative reverse transcription-PCR (qRT-PCR) was performed. Intriguingly, there was no observed significant difference in transcript levels (Fig. 4B). These results suggest that AlaRS levels are posttranscriptionally elevated in the absence of AlaRS proofreading.

As the steady-state analysis is influenced by both the rate of protein decay and active translation, it was of interest to know if the rate of AlaRS decay was modulated by changes in AlaRS fidelity. Translation was stopped in actively growing E. coli cultures by the addition of chloramphenicol, and AlaRS protein levels were monitored over time. One hour post-antibiotic treatment, ∼70% of the AlaRS protein had decayed in the wild-type strain compared to ∼30% in the error-prone AlaRS C666A variant (Fig. 4C). To determine if changes in protein stability are caused by the C666A substitution, *in vitro* active-site stability and *in vitro* thermal melting assays were performed on recombinant protein, and no change in protein stability was observed (Fig. S1 and S2). While a direct mechanism for the elevated and stabilized AlaRS levels remains unclear, contribution by other protein factors which were also dysregulated are predicted to play a role in this effect. Two such factors that may contribute to the observed AlaRS stability are the DnaK-associated factors, GrpE and the molecular chaperone ClpB, both of which were enriched in our data set at 1.8- and 1.9-fold, respectively (33). These proteins are functionally associated with those which were observed during heat stress in a ribosomal decoding mutant (31).

10.1128/mBio.02921-19.1 FIG S1 The AlaRS C666A protein does not have elevated active-site stability *in vitro. In vitro* active-site stability assays of both wild-type and AlaRS C666A recombinant proteins indicated no inherent difference in protein stability. Download FIG S1, PDF file, 0.1 MB. Copyright © 2019 Kelly et al. 2019 Kelly et al. This content is distributed under the terms of the Creative Commons Attribution 4.0 International license.

10.1128/mBio.02921-19.2 FIG S2 The AlaRS C666A protein does not have elevated thermal stability. Melting curves of recombinant wild-type AlaRS and AlaRS C666A proteins indicated no inherent difference in thermal stability. Download FIG S2, PDF file, 0.2 MB. Copyright © 2019 Kelly et al. 2019 Kelly et al. This content is distributed under the terms of the Creative Commons Attribution 4.0 International license.

### Reduced AlaRS fidelity impairs swimming motility.

**FIG 5:**
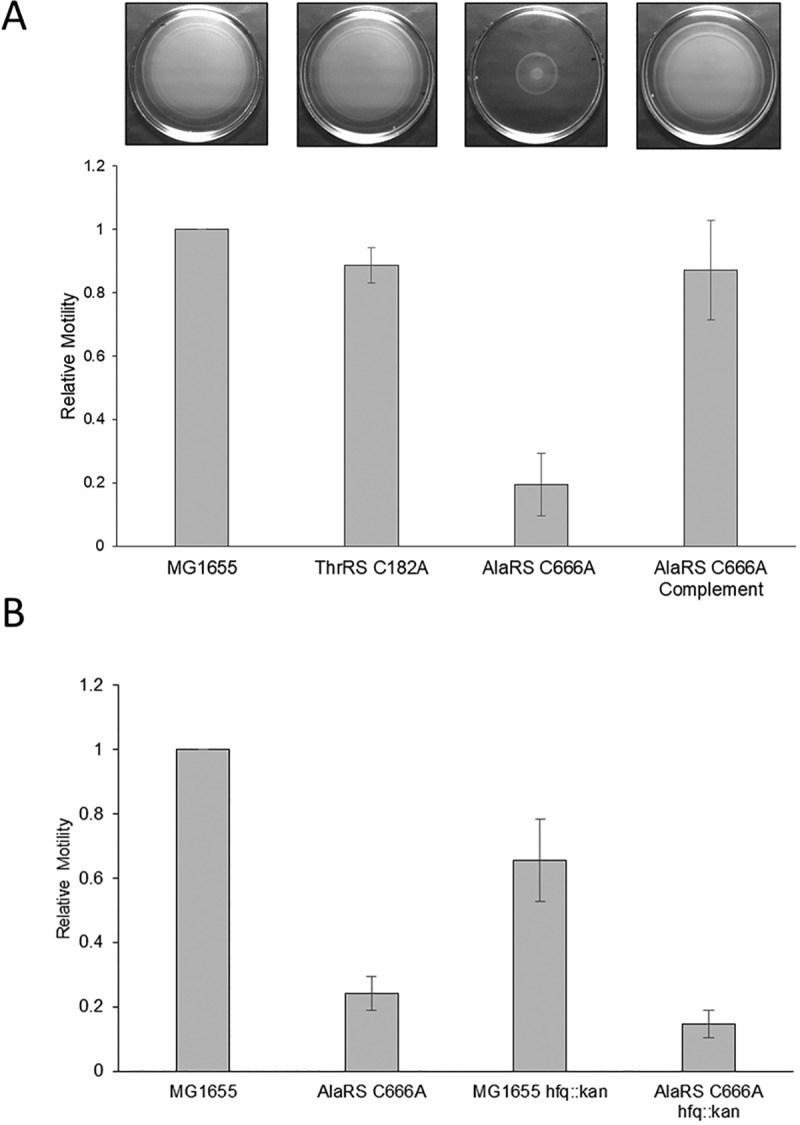
Swimming motility is impaired in the absence of AlaRS fidelity. (A) Representative images (top) and quantification (bottom) of swimming motility data. In the absence of AlaRS proofreading, E. coli has a swimming defect, and this can be rescued by complementation. (B) The observed swimming defect is not exclusively due to small RNA inhibition, as an *hfq* mutant was unable to rescue the defect. All data were plotted relative to a wild-type control for each experiment. Bar graphs shown the averages of the data collected from three experiments, and error bars represent the standard deviation of those experiments.

Aside from changes in metabolism, another notable pathway enriched in the underrepresented proteins was that involved in flagellar assembly, including the master regulator FlhD. Perturbation to swimming motility in response to protein mistranslation has previously been observed in a ribosomal decoding mutant (34). To determine if the underrepresentation of flagellar assembly proteins led to a change in swimming motility, mistranslating strains were grown on swimming agar plates. As anticipated from the total proteomic data set, loss of AlaRS fidelity led to a decrease in swimming motility (Fig. 5A). The decrease in motility was restored when complemented with the wild-type *alaS* allele. Interestingly, there was no decrease in swimming motility in the ThrRS C182A strain, indicating that not all error-prone strains will lead to changes in motility.

In the previous work that implicated translational fidelity and motility impairment, the authors noted the role of the small RNA DsrA to be responsible for these effects. DsrA activity is normally dependent on the small RNA chaperone Hfq (34). To determine if DsrA or other small RNAs are influencing the swimming phenotype in the AlaRS C666A strain, the assay was repeated in an AlaRS C666A background in which *hfq* was deleted. Deleting *hfq* resulted in an overall decrease in motility in both the wild-type and AlaRS mutant strains (Fig. 5B). This observation was consistent with previous reports that Hfq-dependent small RNAs act as both positive and negative regulators of E. coli motility (35). However, these results do suggest that binding of the small RNA DsrA is likely not sufficient to inhibit flagellar synthesis. Aside from posttranscriptional regulation of flagellar genes by small RNAs, motility is regulated by many other environmental and regulatory factors (reviewed in reference 36). As AlaRS-mediated mistranslation led to gross homeostatic perturbation, identification of a solitary mechanistic event leading to motility impairment may not be feasible.

### AlaRS-mediated mistranslation alters the cell membrane.

**FIG 6:**
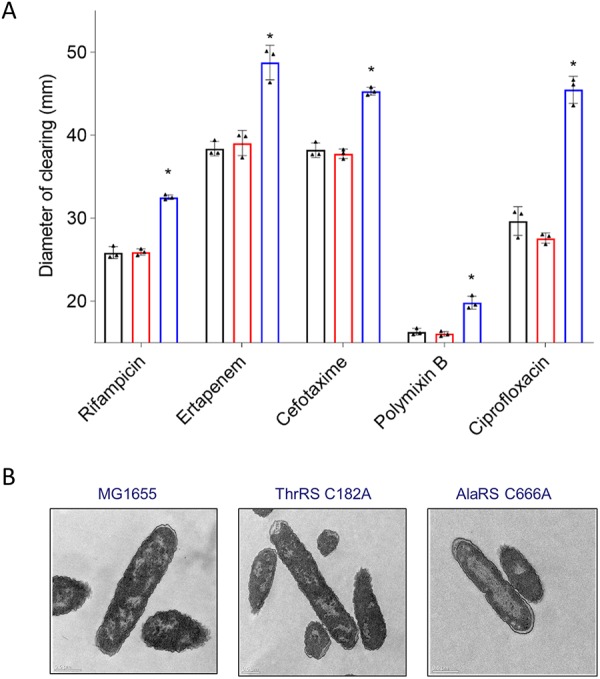
The E. coli membrane is affected when AlaRS fidelity is impaired. (A) The AlaRS C666A mutant (blue) is sensitive to an array of antibiotics, as observed using a disk diffusion assay compared to wild-type (black) and ThrRS C182A (red) E. coli. All experiments were performed in triplicate, with error bars indicating the standard deviation. The asterisks indicate statistical significance as determined using one-way analysis of variance (ANOVA) with Tukey *post hoc* comparison (*P* < 0.05). (B) TEM analysis indicated altered nucleoid morphology in the absence of AlaRS fidelity.

Pathway enrichment during mistranslation also highlighted that fatty acid biosynthesis was perturbed. Furthermore, investigation of stress response activators in the AlaRS C666A proteome indicated that all five envelope stress response regulators were significantly enriched in the error-prone strain, including (fold enrichment) sigma E (2.6×), CpxR (1.3×), RcsB (2.6×), BaeR (3.3×), and PspF (1.7×) (37). It has been suggested that mistranslation of membrane proteins may cause particularly detrimental effects to the cell given the requirement for proper folding and stability across the membrane (38). Together, these observations led to the investigation of membrane integrity in the AlaRS C666A strain. Membrane defects were surveyed using an array of antibiotic sensitivity assays. For all antibiotics screened, the AlaRS C666A strain was significantly more sensitive than wild-type or ThrRS C182A E. coli (Fig. 6A). The observed broad-spectrum sensitivity suggests that higher concentrations of antibiotics are likely able to cross through the cellular envelope rather than there being a targeted sensitivity for the antibiotic mechanism of action. Further support for this hypothesis was the enrichment of the outer membrane porin OmpF, which is known to transport antibiotics across the cell membrane (Data Set S1) (39, 40).

It has also been shown that genetic loci of membrane proteins will spatially coordinate toward cellular membranes, consistent with the transertion model of cotranscriptional and cotranslational processing directly into the inner membrane (41). In addition to the coordination of translation machinery, the transertion model also causes predictive effects on nucleoid dynamics which are disrupted following treatment with transcription or translation inhibitors (42). To determine if there were any changes to ribosome organization in the AlaRS C666A strain, all error-prone strains of interest were subject to transmission electron microscopy (TEM). Under normally growing conditions, there is a heterogeneous distribution of the nucleoid and proteins. However, during times of stress, nucleoid and ribosomal organization will change, leading to noticeable compartmentalization of DNA and ribosomes (42). Following TEM analysis, both the wild-type and ThrRS C182A strains had no discernible patterning of organization with heterogeneous distribution throughout the cellular milieu. In contrast, in the absence of AlaRS proofreading, a clear rearrangement of the cellular nucleoid can be observed, with the ribosomes being sequestered toward the periphery of the cell (Fig. 6B). It remains unclear if this nucleoid rearrangement is solely due to enrichment of the nucleoid-associated proteins or if this is in part due to disruption of the translational machinery.

## DISCUSSION

### AlaRS fidelity is evolutionarily protected.

Across all domains of life, mechanisms have evolved to prevent AlaRS-mediated mistranslation. In addition to endogenous proofreading activity, AlaRS can resample aa-tRNAs, leading to mis-aminoacyl-tRNA hydrolysis. Furthermore, at least three free-standing enzymes, AlaXP (13), Dtd (15), and ANKRD16 in vertebrates (14), have been identified *in vivo* to prevent mischarged tRNA^Ala^ species from accumulating. Additional factors, such as ProXPST1, have also been identified to have proofreading activity *in vitro* against these aa-tRNAs, but their role *in vivo* has yet to be well characterized (43).

Despite the poor specific activity for noncognate aa-tRNA synthesis by AlaRS, results from this work highlight the incredibly high cost of Ala-to-Ser mistranslation in E. coli, which has also been suggested from several studies in higher eukaryotes. The identification of the *sti* allele in mice and its neurodegenerative phenotype provided the first insight into the possible cost of AlaRS-mediated mistranslation in eukaryotes (16). Interestingly, this AlaRS allele only exhibited a minor defect in Ser-tRNA^Ala^ proofreading *in vitro*. Together, these results suggested that low-frequency AlaRS errors are incredibly costly to terminally differentiated neuronal cells. Consistent with that observation, mice homozygous with the AlaRS C723A (corresponding to E. coli C666A) mutation were embryonic lethal (17). While the aforementioned studies observed indicators of elevated protein misfolding, the work described in this report shows global dysregulation of the proteome resulting from a loss of AlaRS proofreading, which may also contribute to the phenotypic defects observed in higher eukaryotes.

### Toxic mistranslation may provide a new target for drug discovery.

As AlaRS-mediated errors led to global changes in proteome regulation, this suggests that tRNA^Ala^ fidelity may act as a key checkpoint for cellular homeostasis. Furthermore, this leads to the possibility of targeting AlaRS fidelity for novel antimicrobial discovery. Given their essential role in protein synthesis, aaRSs have been a promising drug discovery target, with several compounds on the market, including the topical antifungal agent tavaborole. Tavaborole functions by binding to the LeuRS proofreading site, which locks the enzyme in a nonproductive conformation (44). While this drug essentially inactivates the enzyme, the observations from this work suggest that a novel class of aaRS inhibitors could be screened for whose mechanism would block proofreading activity, releasing elevated misaminoacyl tRNA into the cell for translation. As mistranslation has been shown to lead to increased antimicrobial resistance (45), targeting translational fidelity as a monotherapeutic would likely be ineffective. However, chemically inducing mistranslation may act as an agent for chemosensitization that could be exploited for combination therapies with preexisting compounds (46).

### A threshold exists for neutral/beneficial mistranslation events.

Considerable efforts have been devoted to the characterization of beneficial mistranslation events (reviewed in reference 1). The identification of these events leads to several interesting interpretations, some of which have been characterized, including changes in antibiotic resistance (45) and antigen diversity (47). As our understanding of beneficial mistranslation is still in its infancy, the following two questions remained to be explored: can we begin to predict novel beneficial mistranslation events, and do these beneficial mistranslation events provide some consequence to the cell? Evolutionarily, incidences of genomically encoded error-prone aaRSs have been observed across several phyla of intracellular pathogens, including several *Microsporidian* (48, 49) and Mycoplasma (50) species. An interesting observation from these studies is the propensity for the same error-prone aaRSs to arise in these organisms. Of the systems that have been explored, LeuRS, ThrRS, and PheRS have the potential to lose proofreading activity (50). This suggests that errors mediated by these aaRSs are better accommodated by the proteome globally. It is also likely that the proofreading activity of these aaRSs are most easily subject for regulated fidelity, as they bridge the gap between completely degenerate proofreading domains and fully active proofreading function. This is further supported by the high tolerance for PheRS-mediated errors under nutrient-stable conditions in E. coli (51) and the recently observed modulation of Salmonella PheRS fidelity upon oxidative stress (52).

The results from this and other works suggests that due to the high proteotoxic cost of AlaRS-mediated mistranslation, environmentally regulated Ala-to-Ser substitutions will likely not be observed in nature, as the potential benefit (e.g., antigen diversity) does not support the penalty of an inaccurate proteome. This is further supported by the numerous genomically encoded secondary mechanisms to minimize Ala mistranslation, which to date is unique to AlaRS.

In contrast to the requirement for AlaRS fidelity, E. coli ThrRS has recently been shown to lose proofreading activity through at least two different mechanisms, during oxidative stress (19) and posttranslational acetylation (22). Loss of ThrRS proofreading activity is not exclusive to E. coli, as several *Mycoplasma* (50, 53) and yeast (54) species contain ThrRS genes that are naturally proofreading-deficient in cytoplasmic and mitochondrial translation, respectively. Another goal of this work was to determine if sufficiently high levels of Thr-to-Ser mistranslation (i.e., the resulting mistranslation event following ThrRS modification) would be tolerated by E. coli. By generating a chimeric tRNA^Ser^ variant which would translate at Thr codons, it was shown that this mistranslation event does cause growth defects. This suggests that despite the shared terminal functional group, these amino acids are not neutral to the proteome. While it is possible that other factors temporally aligned with native ThrRS modification may influence this result, overall, our findings suggest that not all beneficial mistranslation events are ubiquitously advantageous.

## MATERIALS AND METHODS

### Strain construction and reagents.

To construct new *alaS* and *thrS* E. coli mutants, elements of the “gene gorging” method (55) were combined with other approaches (56) that use I-SceI nuclease to introduce double-strand breaks into the bacterial chromosome to select for cells that have undergone homologous recombination events. The improved method, which will be described in further detail separately, offers the advantage that specific DNA sequence alterations can be made to the chromosome without the need to make any other base pair changes to the DNA. Briefly, PCR products representing the wild-type *alaS* (∼2.7 kbp) and *thrS* (∼1.9 kbp) were amplified from MG1655 (57) genomic DNA and cloned into an R6K-based suicide vector constructed specifically for allelic exchange (58). This mobilizable plasmid encodes resistance to chloramphenicol (Cam) and includes the 18-bp recognition site for the I-SceI nuclease (59). The desired mutations were then introduced into *alaS* (1996TGT→GCG) and *thrS* (1544TGC→GCG) by inverse PCR (60). DNA sequencing was used to confirm that only the desired changes had been made to *alaS* and *thrS* (DNA Facility, Iowa State University, Ames, IA). The resulting plasmids were then transformed into the diaminopimelic acid (DAP) auxotroph donor strain MFD*pir* (61). These transformants were then used as donor strains to introduce the R6K plasmids into MG1655 by conjugation. Cam-resistant (Cam^r^) colonies that grew in the absence of DAP were selected at 37°C, which represented recombinants where the suicide vector had integrated into the E. coli chromosome by a single-crossover event. Cam^r^ recombinants were then transformed with the helper plasmid pSceH, a derivative of pSLTS (56), which expresses the I-SceI nuclease under the control of TetR from a temperature-sensitive pSC101-derivative plasmid and imparts ampicillin (Amp) resistance. Amp-resistant (Amp^r^) transformants were selected at 30°C in the presence of anhydrotetracycline (aTc) to induce I-SceI expression. The surviving colonies were then screened to identify recombinants that had lost the Cam^r^ marker, indicative that the integrated R6K vector had been deleted by a second recombination event. Multiple Cam-susceptible (Cam^s^) recombinants were subsequently tested by PCR and Sanger sequencing to identify mutants that had inherited the new *alaS* and *thrS* alleles.

To determine the relationship between small RNA binding and AlaRS fidelity for swimming motility, *hfq* was deleted in the *alaS* mutant background by P1 transduction of an *hfq*::*kan* allele from NR633 (62). A complete list of strains and plasmids used in this work can be found in Data Set S2.

10.1128/mBio.02921-19.4 DATA SET S2 Strains, plasmids, and primers used in this report. Download Data Set S2, XLSX file, 0.2 MB. Copyright © 2019 Kelly et al. 2019 Kelly et al. This content is distributed under the terms of the Creative Commons Attribution 4.0 International license.

Lysogeny broth (LB) was used for all experiments in rich medium. M9 minimal medium was prepared for all minimal medium experiments. M9 contained 1× M9 salts, 2 g/liter glucose, 1 mg/ml thiamine, 1 mM MgSO_4_, and 0.1 mM CaCl_2_ (63, 64). Amino acid supplementation was performed when indicated. Antibiotic supplementation was performed as follows: kanamycin (Research Products International), 25 μg/ml final concentration; ampicillin (Research Products International), 100 μg/ml final concentration for selection and 20 μg/ml final concentration for mistranslation reporter; and chloramphenicol, 200 μg/ml final concentration to halt translation. Unless otherwise noted, all reagents and oligonucleotides (Data Set S2) were purchased from Sigma-Aldrich.

### Growth analysis.

For all growth experiments, overnight cultures of the respective strains were grown to saturation in either LB or M9 minimal medium with antibiotics supplemented when applicable. Prior to commencing growth analysis, measurements of the optical density at 600 nm (OD_600_) were taken for each saturated culture, and the starting inocula were normalized to an OD_600_ of 0.05. Cultures were grown with aeration at either 37°C or 42°C for heat stress analysis. OD_600_ values were measured at the indicated time points using a CO8000 cell density meter (WPA). The data plotted in Figs 1 and 2 are the averages of at least three biological replicates, with error bars indicating the standard deviation of the replicates.

### Construction of mistranslating tRNA^Ser^ plasmids.

A 195-bp DNA fragment containing the *serW* transcription unit was designed across two partially overlapping synthetic DNA oligonucleotides with 5′ phosphate modifications added. The *serW* gene encodes one of the five serine tRNAs in E. coli (23). The aforementioned oligonucleotides were used in PCR to generate the wild-type full-length *serW* amplicon and subsequently cloned into the pSMART-LCKan blunt cloning vector, following the manufacturer’s recommendations (Lucigen). To generate mistranslating tRNA^Ser^ variants, the pLK-*serW* (Ser) vector was used as the template for site-directed mutagenesis (Stratagene), and the anticodons were mutated to translate at either the alanine or threonine codon. All three *serW* variant plasmids were transformed into MG1655 for growth analysis.

### *In vivo* mistranslation reporter.

It has previously been shown that mutation of an essential serine in β-lactamase (Bla) will inactivate the enzyme (30), which can then be a useful tool for studying missense serine mistranslation (29). The promoter and β-lactamase-encoding gene (*bla*) were amplified from pUC18 and cloned into pSMART-LCKan by blunt ligation (Lucigen). The resulting pLK-Amp vector was then subjected to site-directed mutagenesis to generate the inactive Bla S68A variant. Both the pLK-Amp and pLK-Amp S68A vectors were transformed into MG1655 and MG1655 AlaRS C666A while maintaining selection using the kanamycin resistance cassette on the plasmid.

*In vivo* mistranslation was monitored by streaking MG1655/pLK-Amp/Amp S68A and MG1655 AlaRS C666A/pLK-Amp/Amp S68A on LB plates containing either kanamycin or kanamycin and ampicillin. Plates were grown for 2 days at 37°C to allow sufficient time to observe growth by the MG1655 AlaRS C666A strains.

### Total proteome analysis.

To monitor changes in protein abundance, 20-ml E. coli cultures were grown to an OD_600_ of 1.0 and harvested by centrifugation. The resulting pellet was frozen at –80°C for downstream processing. For cell lysis and protein digestion, cell pellets were thawed on ice, and 2 μl of cell pellet was transferred to a microcentrifuge tube containing 40 μl of lysis buffer (10 mM Tris-HCl [pH 8.6], 10 mM dithiothreitol [DTT], 1 mM EDTA, and 0.5% antilymphocyte serum [ALS]). Cells were lysed by vortexing for 30 s, and disulfide bonds were reduced by incubating the reaction mixture for 30 min at 55°C. The reaction mixture was briefly quenched on ice, and 16 μl of a 60 mM indole-3-acetic acid (IAA) solution was added. Alkylation of cysteines proceeded for 30 min in the dark. Excess IAA was quenched with 14 μl of a 25 mM DTT solution, and the sample was then diluted with 330 μl of 183 mM Tris-HCl buffer (pH 8.0) supplemented with 2 mM CaCl_2_. Proteins were digested overnight using 12 μg of sequencing-grade trypsin. Following digestion, the reaction was then quenched with 12.5 μl of a 20% trifluoroacetic acid (TFA) solution, resulting in a sample pH of <3. The remaining ALS reagent was cleaved for 15 min at room temperature. The sample (∼30 μg protein) was desalted by reverse-phase cleanup using a C_18_ UltraMicroSpin column. The desalted peptides were dried at room temperature in a rotary vacuum centrifuge and reconstituted in 30 μl of 70% formic acid–0.1% TFA (3:8 [vol/vol]) for peptide quantitation by UV280. The sample was diluted to a final concentration of 0.2 μg/μl, and 5 μl (1 μg) was injected for liquid chromatography-tandem mass spectrometry (LC-MS/MS) analysis.

LC-MS/MS was performed using an Acquity ultraperformance liquid chromatography (UPLC) M-Class system (Waters) and Q Exactive Plus mass spectrometer. The analytical column employed was a 65-cm-long, 75-μm-internal-diameter PicoFrit column (New Objective) packed in house to a length of 50 cm with a 1.9-μm ReproSil-Pur 120-Å C_18_-AQ column (Maisch), using methanol as the packing solvent. Peptide separation was achieved using mixtures of 0.1% formic acid in water (solvent A) and 0.1% formic acid in acetonitrile (solvent B) with a 90-min gradient of 0/1, 2/7, 60/24, 65/48, 70/80, 75/80, 80/1, and 90/1 (min/%B, with linear ramping between steps and at a flow rate of 250 nl/min). At least one blank injection (5 μl of 2% B) was performed between samples to eliminate peptide carryover on the analytical column. One hundred femtomoles trypsin-digested bovine serum albumin (BSA) or 100 ng trypsin-digested E. coli wild-type K-12 MG1655 proteins were run periodically between samples as quality control standards.

The mass spectrometer was operated with the following parameters: for MS1, 70,000 resolution, 3e6 AGC target, and *m/z* 300 to 1,700 scan range; for data-dependent MS2, 17,500 resolution, 1e6 AGC target, top 10 mode, *m/z* 1.6 isolation window, 27 normalized collision energy, 90 s of dynamic exclusion, and unassigned and +1 charge exclusion. Data were searched using MaxQuant version 1.6.1.0, with acetyl (N-term), deamidation (NQ), oxidation (M), and phospho (STY) as variable modifications and carbamidomethyl (C) as a fixed modification with up to 3 missed cleavages, 5 amino acids (aa) minimum length, and 1% false-discovery rate (FDR) against the UniProt E. coli database. Searches were analyzed with Perseus version 1.6.2.2.

### Immunoblotting.

Steady-state AlaRS levels were determined by harvesting growing E. coli cultures when they reached an OD_600_ of 0.7. Cell pellets were resuspended in SDS loading dye and boiled for 10 min before equal volumes of total cellular material were loaded on a 10% SDS-polyacrylamide gel and separated by electrophoresis. Proteins were transferred onto a 0.45-μm nitrocellulose membrane (Amersham) before blocking for an hour in 5% milk in Tris-buffered saline with Tween 20 (TBST). E. coli AlaRS (96 kDa) was probed with an anti-E. coli AlaRS antibody (1:1,000 dilution; ProSci custom antibody) and anti-rabbit horseradish peroxidase (anti-HRP; 1:5,000 dilution; GE Healthcare). Membranes were stripped using the Abcam mild stripping protocol and reprobed with an HRP-conjugated anti-sigma70 (70 kDa) antibody (1:4,000 dilution; BioLegend) as a loading control. HRP signals were developed using Clarity electrochemiluminescence (ECL) substrate (Bio-Rad) and monitored using ChemiDoc and the accompanying software (Bio-Rad). Western blot densitometric quantification was performed using the ImageJ software (65).

Protein stability assays were performed essentially as described above, with minor modifications. Cells were grown to an OD_600_ of 0.5, and 1 ml of cells was removed, pelleted, and frozen as the time zero (T_0_) sample. Simultaneously, chloramphenicol was added to the remaining cultures (200 μg/ml final concentration) and continued to grow. At the indicated time points, samples were removed, pelleted, and frozen until all samples were collected. To quantify the relative protein stability, densitometric analysis was performed and normalized to the abundance of protein at T_0_ for a given biological replicate. The data plotted are the average stability of three independent biological replicates, with error bars indicating the standard deviation of the replicates.

### Transcript analysis.

From saturated overnight cultures, strains were normalized and back diluted, and cultures were grown to an OD_600_ of 0.7 prior to pelleting. Bacterial pellets were resuspended in RNA*later* (Ambion) and stored at 4°C overnight. The pelleted cellular material was resuspended in buffer containing 20 mM sodium acetate (pH 5.2), 1% SDS, and 0.3 M sucrose and extracted in equal-volume acid phenol chloroform at 65°C. The aqueous phase was subsequently reextracted with acid-phenol chloroform at room temperature before one final chloroform extraction. RNA was precipitated in 1 volume isopropanol and 1/10 volume sodium acetate. DNA was removed from samples using Turbo DNase (Invitrogen), and RNA was extracted using acid-phenol chloroform prior to ethanol precipitation. Reverse transcription was performed using 100 ng RNA and SuperScript IV (Invitrogen), following the manufacturer’s recommendations. Transcript abundance was determined using primers specific for target mRNAs and SsoAdvanced Universal SYBR green supermix (Bio-Rad), and target mRNAs were analyzed using a CFX96 real-time PCR detection system (Bio-Rad). Data were analyzed using the Pfaffl method (66) and are the result of three technical replicates from three independent biological replicates.

### Recombinant AlaRS purification.

The effect of the AlaRS C666A substitution on protein structure was determined by monitoring the stability of recombinant protein. Wild-type or mutant *alaS* genes were amplified from their respective E. coli strains and cloned into pET21b at NdeI and XhoI restriction cloning sites. The resulting expression construct generated an in-frame C-terminal His tag for metal affinity purification. AlaRS proteins were expressed in BL21(DE3) after growing cells to at 37°C to mid-log phase and subsequently inducing expression with 500 μM isopropyl-β-d-thiogalactopyranoside (IPTG) for 4 h. Harvested cells were resuspended in buffer A (50 mM Tris-HCl [pH 8.0], 300 mM NaCl, and 10 mM imidazole) and EDTA-free cOmplete mini protease inhibitor (Sigma) prior to lysis by sonication. Clarified lysate was passed over a Talon metal affinity column (TaKaRa), washed with buffer A, and finally eluted with buffer B (50 mM Tris-HCl [pH 8.0], 300 mM NaCl, and 250 mM imidazole). Proteins were dialyzed in two stages to remove imidazole and to store the enzyme in 50% glycerol.

Initial enzyme concentrations were determined by active-site titration (67). Briefly, enzyme was incubated with 8 mM ATP, 150 μM [^14^C]alanine (PerkinElmer), pyrophosphatase, and 1× buffer (100 μM HEPES [pH 7.2], 30 mM KCl, and 10 mM MgCl_2_). The reaction mixtures were incubated at 37°C for 20 min before filtering through a Protran BA 45 nitrocellulose membrane (Whatman). Filter disks were prewashed with 0.5× buffer and subsequently washed three times with 0.5× buffer after sample filtration. The disks were dried, and radiolabeled signal was quantified using scintillation counting.

### Active site and thermal stability.

To monitor changes in protein stability, active-site titration was performed as described above using 5 μM enzyme before (T_0_) and after (T_60_) incubating the enzyme at 37°C for 60 min prior to analysis. The stability of the enzyme was determined by plotting the change in activity after incubation at 37°C (activity at T_60_/activity at T_0_) (68).

Changes in protein stability were also monitored using circular dichroism (CD). Wild-type and mutant proteins were resuspended 100 mM potassium dihydrogen phosphate and transferred to a 0.5-ml Amicon Ultra centrifugal filter tube. Both proteins were then centrifuged at 10,000 × *g* for 15 min at 4°C to remove Tris and glycerol, which are incompatible with CD analysis. The centrifugation was repeated three times with the addition of 200 μl of 100 mM potassium dihydrogen phosphate after each spin. The concentration was determined using a NanoDrop spectrophotometer, and a final concentration of 0.5 mg/ml was used for the CD experiments.

Variable temperature measurement was performed on the Jasco J-815 circular dichroism spectrometer, where the change in molar ellipticity of the protein was measured as a function of temperature to determine the melting temperature (*T_m_*). The molar ellipticity was measured at 222 nm, which captures the extent of alpha helicity present in the protein. A range of temperatures from 25°C to 95°C was selected. The values of molar ellipticity obtained were converted to fraction folded using the following equation:

α = (θT-θU)/(θF-θU)

where α is the fraction of folded protein, θ_T_ is the molar ellipticity at 222 nm at any temperature, θ_F_ is the molar ellipticity at 222 nm of the completely folded form (at 25°C), and θ_U_ is the molar ellipticity at 222 nm of the completely unfolded form (at 95°C). The fraction folded versus temperature was plotted, where the *T_m_*, the temperature at which 50% of the protein is folded, was estimated.

### Swimming motility.

Swimming plates were prepared in LB broth, as described above, and 0.2% agar. From overnight saturated cultures, cells were normalized to an OD_600_ of 0.5, and 5 μl of cells was spotted on freshly solidified swimming plates. Plates were incubated at 37°C for 4 to 8 h prior to imaging. Swimming distance was calculated using ImageJ, and the relative swimming distance was determined by comparing the mutant genotype of interest to a wild-type control. Bar graphs in Fig 5 indicate the average relative motility for three independent biological replicates, and error bars indicate the standard deviation of the replicates.

### Antibiotic sensitivity.

Saturated overnight cultures were struck on LB agar plates with cotton swabs, creating a bacterial lawn. Prior to culturing at 37°C, an array of antimicrobial sensitivity disks (Oxoid) were placed on the bacterial lawn. Following the overnight incubation, plates were imaged, and the distance of disk diffusion was measured using ImageJ.

### Transmission electron microscopy.

Strains were back diluted from a saturated overnight culture and grown to an OD_600_ of 0.8. Cells were gently pelleted at 3,000 rpm for 5 min. Pellets were prefixed in 4% electron microscopy (EM)-grade buffered formaldehyde for shipping and storage. Bacterial pellets were subsequently fixed in 2.5% glutaraldehyde–0.1 M phosphate buffer at 4°C. Pellets were then washed three times for 5 min in 0.1 M phosphate buffer, placed in 1% aqueous osmium tetroxide for 90 min, and then reduced with ferrocyanide for 60 min at room temperature. Pellets were washed three times with water, dehydrated in a graded acetone series, and embedded in Spurr’s resin. Sections were cut to 60 nm thin, collected on Formvar-filmed copper grids, and stained with 2% uranyl acetate and Reynold’s lead citrate. Bacterial sections were imaged at 80 kV in a Zeiss EM10 using a Gatan Erlangshen charge-coupled-device (CCD) camera.
