# Supplementary material for: Alanyl-tRNA Synthetase Quality Control Prevents Global Dysregulation of the Escherichia coli Proteome
Source: mBio. 2019 Dec 17;10(6):e02921-19. doi: 10.1128/mBio.02921-19 (PMC6918089; doi:10.1128/mBio.02921-19)
Supplement: FIG S1 [file mBio.02921-19-sf001.pdf]

Supplemental Figure 1.

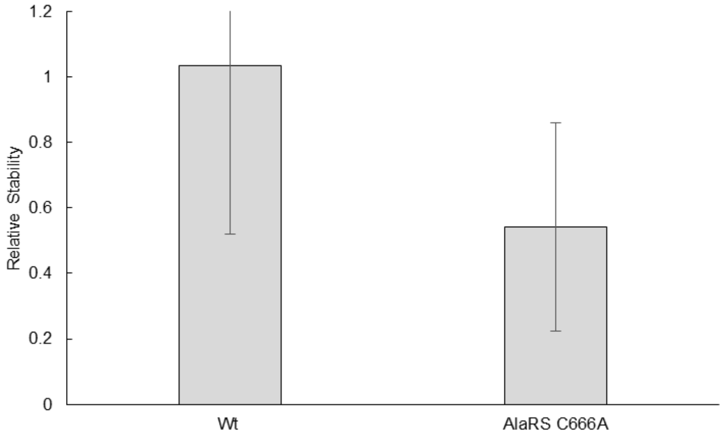

Supplemental Figure 1. *The AlaRS C666A protein does not have elevated active site stability in vitro. In vitro* active site stability assays of both wild-type and AlaRS C666A recombinant proteins indicated no inherent difference in protein stability.
