# Supplementary material for: Alanyl-tRNA Synthetase Quality Control Prevents Global Dysregulation of the Escherichia coli Proteome
Source: mBio. 2019 Dec 17;10(6):e02921-19. doi: 10.1128/mBio.02921-19 (PMC6918089; doi:10.1128/mBio.02921-19)
Supplement: FIG S2 [file mBio.02921-19-sf002.pdf]

Supplemental Figure 2.

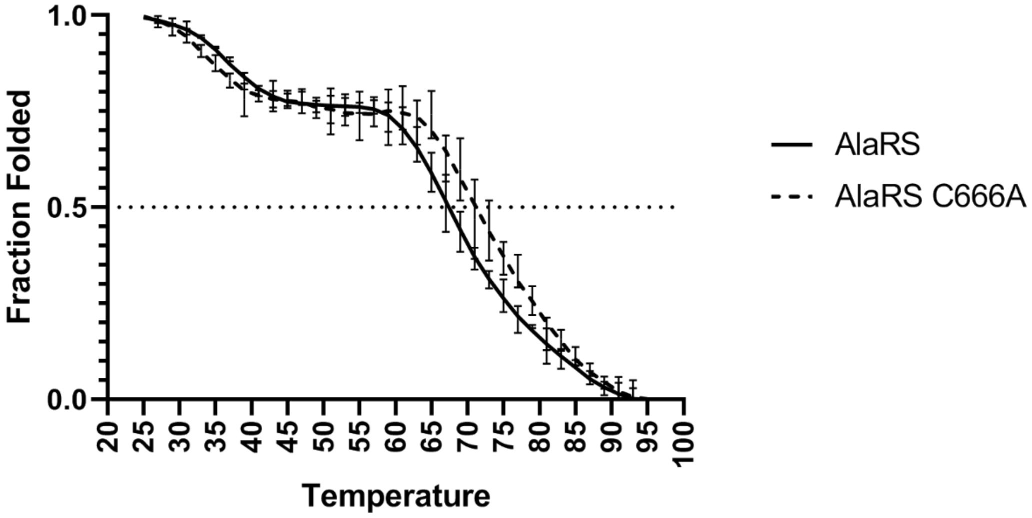

Supplemental Figure 2. *The AlaRS C666A protein does not have elevated thermal stability.* Melting curves of recombinant wild-type AlaRS and AlaRS C666A proteins indicated no inherent difference in thermal stability.
